# Ascending Mount Orizaba

**Published:** 1892-12

**Authors:** 


### ASCENDING MOUNT ORIZABA.

The first ascent of the extinct volcano, Orizaba, in Mexico, was made by Baron Muller, in 1856. The baron and his friends climbed with great difficulty nearly to the top. They had been deserted by their guides, and were so exhausted that they could go only a few feet without stopping to rest. Suddenly they discovered that they were separated by thin ice only from a vast abyss. With the utmost caution they retreated to firmer ground, losing their provisions as they did so. A blinding snow storm obliged them to camp for the night. In the morning two of the party were stricken with temporary blindness, and a descent was made to the village San Andres Chalchicomula.

The baron was not daunted. He started with another party to ascend another side of the mountain.

My caravan, he writes, consisted of Mr. Campbell, M. de La Huerta and two Indian guides, all on horseback. We picked our way among the rocks by dangerous and apparently impossible paths. Once Heurta fell with his horse. He was on a narrow, slippery rock, and I expected to see him go over the precipice, but his Mexican horse saved himself with wonderful quickness and skill. Late that night we camped in a cave.

Early next morning we reached the zone of perpetual snow. The air was so rarefied that the horses could hardly breathe, they became exhausted, and had to be sent back to the cave. At noon we rested and lunched on a little snow platform. It was the last surface of the kind below the summit. One of our obstacles was the softness of the snow; at every step we sank to the knee. We were ascending at an angle of forty-five degrees, and often had to crawl on our hands and knees.

In spite of a veil and colored glasses my eyes were almost blinded. The air was so rarefied that we could hardly breathe. At every breath sharp pains cut through my lungs, and I fainted every few minutes. The sun became clouded, and we began to be terribly cold. Often we came to a perpendicular wall of snow, and had to go around it with infinite toil and care.

Late in the day the summit was still distant. The guides refused to go further, and my friends lost courage; but I determined to proceed, with or without them, and they decided to go on. A fine, icy snow began to fall, and we were chilled to the bone.

At a quarter to six o'clock, after great exertions, almost utterly exhausted, we reached the edge of the crater. I soon recovered from a fainting fit, and made the observations which I wished to have as to the shape, size and appearance of the crater. My estimate of the height of Orizaba is 5,527 metres.

The Indian guides made sleds of straw mats, and we descended on them. This was more like a fall in mid air than anything else. In a few minutes we covered a space that it had taken us five hours to climb. At half-past nine we reached the cave and camped for the night, after fourteen hours of hard work.

The next day, as we approached San Andres Chalchicomula, we were surprised to see the entire population of the town, with music and banners, coming to meet us. One of the guides had gone on ahead and reported our success.

